# Safety profile of hydroxychloroquine used off‐label for the treatment of patients with COVID‐19: A descriptive study based on EudraVigilance data

**DOI:** 10.1111/fcp.12797

**Published:** 2022-05-13

**Authors:** Domenico Motola, Giulia Bonaldo, Nicola Montanaro

**Affiliations:** ^1^ Unit of Pharmacology, Department of Medical and Surgical Sciences Alma Mater Studiorum University of Bologna Bologna; ^2^ Alma Mater Studiorum University of Bologna Bologna Italy

**Keywords:** benefit–risk balance, COVID‐19, hydroxychloroquine, off‐label, safety

## Abstract

At the beginning of the COVID‐19 pandemic, worldwide attempts were made to identify potential drugs effective against the COVID‐19. Hydroxychloroquine was among the first receiving attention. However, following its use in therapy, it has been shown that hydroxychloroquine was not only ineffective but probably, due to its known side effects, even responsible of increased mortality of patients. The objective of this study was to review the safety profile of hydroxychloroquine used off‐label for the treatment of COVID‐19. We analyze the reports of suspected adverse drug reactions (ADRs) collected in EudraVigilance, the European database of ADR reports. We collected 2266 reports for 2019 and 6525 for 2020. The most reported ADRs during 2020 were those relating to cardiac, hepatic, renal toxicity such as QT prolongation with 400 cases in 2020 (of which, 345 cases—9.97%—with COVID‐19 as a therapeutic indication) versus 1 case only in 2019 (0.01%), long QT syndrome: 38 cases in 2020 (36 as COVID‐19 treatment) versus 0 in 2019, hepatitis: 13 cases in 2019 (0.11%) and 132 in 2020, and 32 cases (24, 0.69%) of acute kidney injury in 2020 and only 3 cases in 2019. Moreover, some important vision‐related ADRs also increased significantly during 2020, such as retinal toxicity with 92 cases in 2020 versus 7 in 2019. Even though with its intrinsic limitations, our results may be added to the most recent scientific evidence to confirm the unfavorable risk profile of hydroxychloroquine in its off‐label use in the treatment of COVID‐19 disease.

**List of Abbreviations**
ADR: adverse drug reaction
COVID‐19: coronavirus disease 2019
DMARD: disease modifying antirheumatic drugs
NSAID: nonsteroidal anti‐inflammatory drugs

## 1 BACKGROUND

The rapid outbreak of coronavirus disease 2019 (COVID‐19), that is, the severe acute respiratory syndrome due to coronavirus 2 (SARS‐CoV‐2) in late 2019 and early 2020, has caused an unprecedented situation for patients and clinicians. National health services and hospitals of the countries mainly affected by the pandemic have been severely challenged. At the same time, a desperate race began to identify drugs that could limit patient pressure on hospitals and that could reduce the impact of this illness on mortality. Hydroxychloroquine was among the first drugs receiving attention as possible therapy, on the basis of very preliminary laboratory data and misleading evidence from flawed but heavily publicized studies [1, 2, 3]. Owing to the endorsement of unprepared politicians and media [4, 5], hydroxychloroquine became in a few days the life‐saving drug for the early phases of the pandemic. Hydroxychloroquine is an old chemically synthesized drug with a negligible cost, originally used as an antimalarial and subsequently for some chronic inflammatory diseases. Compared to chloroquine, the hydroxylated derivative presented a lower risk of serious side effects [6]. In its use against COVID‐19 disease, the scientific evidence coming from ad hoc clinical studies subsequently showed that hydroxychloroquine was not only ineffective but probably, due to its known and often serious side effects, even responsible of increased mortality of patients [7, 8, 9, 10, 11]. To make matters worse, hydroxychloroquine has been prescribed off‐label in association with azithromycin (antibacterial drug belonging to the class of macrolides), which has long been known for being associated with cardiovascular adverse effects, especially in elderly individuals [12, 13]. Also for the latter, subsequent evidence has ruled out any efficacy in the treatment of the various forms of COVID‐19 infection [14, 15]. The aim of the present research was to review the safety profile of hydroxychloroquine used off‐label for the treatment of COVID‐19 infection using the reports of suspected adverse drug reactions collected in Europe during 2020.

## 2 METHODS

Data were downloaded from the European database of suspected adverse drug reaction reports (EudraVigilance) using the online interface adrreports.eu (EudraVigilance—European database of suspected adverse drug reaction reports. Available at: http://www.adrreports.eu/). EudraVigilance is the system for managing and analyzing all the reports of suspected adverse drug reaction (ADR) collected in the European Union.

We focused on all the reports of ADR having hydroxychloroquine as a suspected drug (i.e., the drug associated with the ADR as determined by the initial reporter), sent in the years 2019 and 2020.

The information analyzed was unique EU local  number, report type, EV receipt date, primary source qualification (healthcare professional or not), primary source country for regulatory purpose (European or non‐European economic area), literature reference (if available), patient age group (0–1 month, 2 months to 2 years, 3–11 years, 12–17 years, 18–64 years, 65–85 years, >85 years, or not specified), patient sex (female, male, or not specified), the seriousness of ADRs, and reaction list preferred term (PT). The PT is a distinct descriptor for a symptom, sign, disease diagnosis, therapeutic indication, investigation, surgical or medical procedure, and medical social or family history characteristic (https://www.meddra.org/how-to-use/basics/hierarchy). An analysis on concomitant drugs was also done. The analysis was carried out by drug–reaction pair, that is, a suspected ADR report may concern only one drug and one ADR or one drug and several ADRs; therefore, the analysis is performed for each drug–reaction pair generated by the report.

We performed a descriptive analysis of the number and frequencies of drug–reaction pairs when hydroxychloroquine was reported as suspected drug. Demographic characteristics and other information (type of reporters, source of countries, seriousness, outcome of the ADRs limited to death, etc.) were also provided. A chi‐square test (or a Fisher's exact test) was used to assess the statistical significance of the differences among between 2019 and 2020 numbers of reports.

Since in 2020 hydroxychloroquine was used as off‐label post‐exposure prophylaxis or treatment of COVID‐19, data for 2019 were employed as a comparison to highlight possible differences in size and type ADR pattern in relation to the different use of the drug between the two periods.

## 3 RESULTS

**FIGURE 1:**
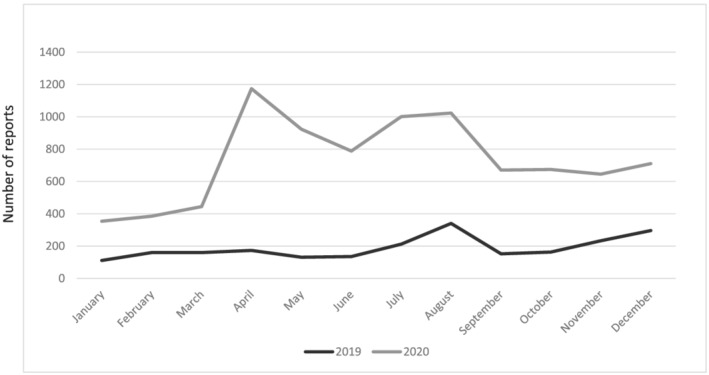
Monthly distribution of hydroxychloroquine ADR reports in 2019 and 2020

In 2019, 2266 reports of ADRs having hydroxychloroquine as suspected drugs were submitted to the EudraVigilance network with a monthly distribution as shown in Figure 1. In 2020, the number of reports increased to 6525 (+188% as compared to 2019). The majority of reports in the 2 years under analysis came from health professionals, 78.02% in 2019 and 84.37% in 2020. Twenty‐three percent of reports (524 out of 2266) came from European economic area in 2019 and 26% (1704 out of 6525) in 2020. Forty‐one percent (919 out of 2266) had no concomitant drugs in 2019. The most reported concomitant drugs were tofacitinib, leflunomide, and other DMARDs, ciclosporin, methotrexate, corticosteroids (prednisone and prednisolone), and several NSAIDs. In 2020, 3079 reports out of 6525 had no concomitant drugs (47%), whereas the most reported concomitant drugs, in addition to the previous ones, were azithromycin (listed in 51 reports in 2020 vs. 2 in 2019), tocilizumab (in 74 reports in 2020 vs. 17 in 2019) and ritonavir/lopinavir (in 62 reports in 2020 vs. 0 in 2019), dexamethasone (in 36 reports in 2020 vs. 3 in 2019), anakinra (33 in 2020 vs. 3 in 2019), and ibuprofen (85 in 2020 vs. 23 in 2019). Table 1 shows the distribution of reports for gender and age classes: Most of reports for both 2019 and 2020 concerned females with a similar distribution in the age classes, whereas in 2020, the number of reports for males was significantly higher compared to 2019: 1597 reports out of 6525 (24.48%) in 2020 versus 293 out of 2266 (12.93%) in 2019 (*P* < 0.01). Most of 2020 male reports were observed for the age classes 18–64 (11.71% vs. 5.16% in 2019) and 65–85 years (7.45% vs. 2.60% in 2019).

**TABLE 1:** Distribution of reports for gender and age classes

	2019	%	2020	%
Female	1600	70.61	4604	70.56
0–1 month	6	0.26	9	0.14
2 months to 2 years	3	0.13	4	0.06
3–11 years	2	0.09	36	0.55
12–17 years	9	0.40	60	0.92
18–64 years	726	32.04	2244	34.39
65–85 years	290	12.80	769	11.79
More than 85 years	13	0.57	58	0.89
Not available	551	24.32	1424	21.82
Male	293	12.93	1597	24.48
0–1 month	1	0.04	4	0.06
2 months to 2 years	2	0.09	5	0.08
3–11 years	2	0.09	7	0.11
12–17 years	1	0.04	13	0.20
18–64 years	117	5.16	764	11.71
65–85 years	59	2.60	486	7.45
More than 85 years	5	0.22	35	0.54
Not available	106	4.68	283	4.34
Gender not available	128	5.65	324	4.97
3–11 years	0	0.00	1	0.02
12–17 years	0	0.00	1	0.02
18–64 years	7	0.31	20	0.31
65–85 years	2	0.09	7	0.11
More than 85 years	0	0.00	2	0.03
Not available	119	5.25	293	4.49
No data available	245	10.81	0	0.00
Total	2266	100.00	6525	100.00

Overall, the 2266 reports collected in 2019 corresponded to 12 032 drug–reaction pairs whereas those reported in 2020 generated 44 770 drug–reaction pairs.

Table 2 lists the most reported ADRs during 2020 (with specific data for those ADRs received during 2020 with “COVID‐19” as explicit therapeutic use) as compared to 2019. With the exception of the reports regarding off‐label use and therapeutic failure (which also increased enormously compared to 2019), the most significant ADRs were those relating to cardiac, hepatic, renal toxicity and impaired vision. Among the cardiovascular ADRs, the most significant ones were those related to QT prolongation with 400 cases in 2020 (of which, 345 cases—9.97%—with COVID‐19 as a therapeutic indication) versus 1 case only in 2019 (0.01%), long QT syndrome with 38 cases in 2020 (36, 1.04% as COVID‐19 treatment) versus 0 in 2019, atrial fibrillation with 20 cases (16, 0.46%) versus 1 in 2019, cardiac arrest with 35 cases (17, 0.49%) versus 2 in 2019, and others. A similar trend was observed in the case of hepatic ADRs: For example, hepatitis were 13 cases in 2019 (0.11%) whereas 132 (79, 2.28% in COVID‐19 patients) in 2020, hepatocellular injury from 6 cases in 2019 to 43 (39, 1.13%) in 2020, cholestasis, 1 case in 2019 versus 38 (37, 1.07%) in 2020, and so on for other adverse reactions affecting the liver. With regard to renal ADRs, 32 cases (24, 0.69%) of acute kidney injury in 2020 should be highlighted as compared to only 3 cases reported in 2019, and for the ADR renal failure, 21 cases were reported in 2019 versus 62 (8, 0.23%) in 2020. Moreover, some important vision‐related ADRs also increased significantly during 2020, such as retinal toxicity with 92 cases in 2020 versus 7 in 2019 and visual impairment with 96 cases in 2020 versus 55 in 2019. In addition, 10 cases of rhabdomyolysis reported in 2020 (9 for off‐label use in patients with COVID‐19) against no cases in 2019, as well as the 18 cases of hemolytic anemia in 2020 (15, 0.43% in patients with COVID‐19) towards no case in 2019. Table 3 lists the breakdown of the ADR reports according to their seriousness (death, life threatening, cause or prolongation of hospitalization, and other important medical condition) in 2019 versus 2020. In 2019, 1756 reports were classified as serious (corresponding to 77.49%), and among them, 26 cases had a fatal outcome. In 2020, 5800 reports were serious (corresponding to 88.89%) of which 223 with a fatal outcome. Serious reports for death, life threatening, and causes/prolonged hospitalization in 2019 were significantly higher in 2020 (*P* < 0.01).

**TABLE 2:** Most reported ADRs for hydroxychloroquine during 2020 as compared to 2019

ADR	2020		2019		*P* value^b^
	*N* ^a^	%^a^	*N*	%	
Off‐label use	1415 (522)	3.16 (15.09)	161	1.34	<0.01
Electrocardiogram QT prolonged	400 (345)	0.89 (9.97)	1	0.01	<0.01
Long QT syndrome	38 (36)	0.08 (1.04)	0	0.0	<0.01^c^
Hepatic enzyme increased	256 (26)	0.57 (0.75)	56	0.47	<0.01
Product use in unapproved indication	158 (50)	0.35 (1.45)	20	0.17	<0.01
Hepatitis	132 (79)	0.29 (2.28)	13	0.11	<0.01
Visual impairment	96 (2)	0.21 (0.06)	55	0.46	<0.01
Retinal toxicity	92 (0)	0.21 (0.0)	7	0.06	<0.01
Hepatocellular injury	43 (39)	0.1 (1.13)	6	0.05	<0.05
Acute kidney injury	32 (24)	0.07 (0.69)	3	0.02	<0.05
Renal failure	62 (8)	0.14 (0.23)	21	0.17	N.S.
Myocardial infarction	51 (2)	0.11 (0.06)	18	0.15	N.S.
Drug‐induced liver injury	48 (4)	0.11 (0.12)	9	0.07	N.S.
Urinary tract infection	47 (1)	0.1 (0.03)	26	0.22	N.S.
Cardiac failure	41 (5)	0.09 (0.14)	2	0.02	<0.01
Cardiac arrest	35 (17)	0.08 (0.49)	2	0.02	<0.01
Liver injury	31 (13)	0.07 (0.38)	1	0.01	<0.01
Atrial fibrillation	28 (16)	0.06 (0.46)	1	0.01	<0.05
Hypertransaminasemia	27 (26)	0.06 (0.75)	0	0	<0.01^c^
Hemolysis	12 (10)	0.03 (0.29)	0	0.0	<0.05^c^
Hemolytic anemia	18 (15)	0.04 (0.43)	0	0.0	<0.01
Cholestasis	38 (37)	0.08 (1.07)	1	0.01	<0.01
Rhabdomyolysis	10 (9)	0.02 (0.26)	0	0.0	N.S.

^a^ 2020 ADRs reported with “COVID‐19” as therapeutic use.

^b^ Chi‐square test.

^c^ Fischer exact test.

**TABLE 3:** Seriousness of ADRs for hydroxychloroquine in 2020 as compared to 2019

ADR	2020		2019		*P* value^a^
	*N*	%	*N*	%	
Other medically important condition	4313	66.10	1470	1.34	N.S.
Life threatening	239	3.66	16	0.01	<0.01
Caused/prolonged hospitalization	1025	15.71	244	0.0	<0.01
Death	223	3.42	26	0.47	<0.01
None/unknown	725	11.11	510	0.17	<0.01
Total	6525	100	2266	100	

^a^ Chi‐square test.

Among the fatal cases in 2019, hydroxychloroquine was used for patients with rheumatoid arthritis, systemic lupus erythematosus, and collagen diseases of for unknown indications, whereas in 2020, it was used also for patients with COVID‐19 (53 cases). Among the causes of death most frequently identified in the 53 reports of 2020, we found cardiovascular causes (arrhythmia, cardiac arrest, cardiac failure, electrocardiogram QT prolonged, and others), renal causes (renal failure, acute kidney injury) and others (hemolytic anemia).

## 4 DISCUSSION

The present analysis of the European pharmacovigilance data concerning hydroxychloroquine has shown a relevant increase of the overall ADR reporting in the period in which the drug has been used also as off‐label treatment in patients infected with COVID‐19 in the absence of even minimal data of clinical efficacy. The greatest increase was observed from April 2020 (which represents the worst period of the first wave of the pandemic) until August 2020, that is, when the published scientific evidence leads to abandon the use of this drug for COVID‐19 infection.

The benefit–risk balance of hydroxychloroquine in its approved therapeutic indications was considered favorable but critical, especially for its well‐known cardiovascular and hepatic adverse effects (it was authorized in Italy for the first time on May 19, 1959, https://farmaci.agenziafarmaco.gov.it/aifa/servlet/PdfDownloadServlet?pdfFileName=footer_008055_013967_RCP.pdf&sys=m0b1l3). In its off‐label use for the treatment of COVID‐19 patients, the risk–benefit balance of the drug is absolutely unfavorable, and this clinical use should be no longer allowed. The data of the present study show a considerable increase of serious and life‐threatening adverse reactions such as QT prolongation, renal and hepatic damage, rhabdomyolysis, and also other less serious ADR impairing the quality of life, such as hydroxychloroquine‐associated visual disorders. For visual disturbances, on the one hand, these ADRs are known to occur mainly with chronic use of the drug; however, as noted by Melles and Marmor, hydroxychloroquine retinopathy is more common than previously recognized, and the risk may be much higher because retinopathies can be detected earlier when using more sensitive screening techniques [16]. As for the known arrhythmic risk of hydroxychloroquine, a much higher caution should have been used in the concomitant use of other QT‐prolonging drugs such as azithromycin [17], which instead has been often added to hydroxychloroquine in patients with COVID‐19, who were also suffering from additional risk factors, such as elderly age with underlying heart disease, electrolyte imbalance, systemic inflammatory process, and myocardial injury [18, 19]. Among the 51 cases of concomitant use of azithromycin that we observed among COVID patients, heart rhythm disturbances (electrocardiogram QT prolonged, long QT syndrome, arrhythmia, bradycardia, and others) were reported in 17 cases.

Moreover, several guidelines and publications had rapidly warned against this specific risk and suggested practical measures (e.g., daily electrocardiographic monitoring) for its mitigation [20, 21]. By a stricter application of those data, many ADR involving hydroxychloroquine might have been prevented.

Our data on the increase of reports of cardiac ADR are in accordance with those of Rosenberg et al. [14] who observed that cardiac arrest was more frequent in patients who received hydroxychloroquine with azithromycin, compared with no exposure. Many other manuscripts have come to the same conclusions [22, 23]. The increased cases of QT prolongation could have caused the higher mortality rate in these patients without any benefit, as shown in the meta‐analysis by Axfors et al. [5].

Regarding renal ADRs of hydroxychloroquine (acute renal injury and renal failure), these are not listed in the summary of product characteristics of the drug‐based medicinal products. Concomitance of rhabdomyolysis or hemolytic anemia in our cases of renal ADRs were low. The data indicate that rhabdomyolysis was reported in two cases of acute kidney injury in 2020, and hemolytic anemia was also present in four cases of acute kidney damage and in two cases of renal failure. Thus, both rhabdomyolysis and hemolytic anemia may explain some cases but not most of reports of kidney damage in 2020. Some authors suggest that the probable mechanism involves accelerated apoptosis and inhibited proliferation of proximal tubular epithelial cells via autophagy–lysosomal pathway disruption and senescence promotion [24]. Given the relevance of these ADRs, regulatory bodies should include kidney damage as a possible ADR resulting from post‐marketing observations.

The interpretation of the results of the present study suffers of the usual limitations affecting spontaneous reporting. As for the quality of the data, the information reported might be not in‐depth enough to allow a correct and complete comprehension of the cases. Moreover, the absence of a denominator (i.e., number of patients exposed to off‐label use of hydroxychloroquine) does not allow an estimate of the real frequency of the events. Lastly, there are many possible confounders to be considered such as concomitant therapies, predisposing conditions and other risk factors.

On the other side, the clinician during the off‐label use of a drug may pay more attention on the early identification of possible adverse effects.

Hydroxychloroquine, with or without azithromycin, has been considered as a possible therapeutic option for patients with COVID‐2019 on the basis of preliminary and anecdotal data. In terms of clinical evidence, no elements were available to recommend the use of this drug in the treatment of COVID‐19. In fact, clinical data demonstrated that hydroxychloroquine did not reduce mortality and the duration of mechanical ventilation but only increase the risk of heart rhythm problems, blood and lymph disorders, kidney injury, liver problems, and failure (4, 5, and 7), and it is quite clear that such choice to use this drug anyway was founded on an extra scientific approach [25], although partially justified by the fact that both patients and clinicians were devoid of any specific treatment. However, this phenomenon has undermined the structured approach with which drugs should be evaluated before entering the clinical use [26].

Hydroxychloroquine, even if now abandoned by the hospital doctors for the treatment of COVID‐19, is still largely present as alternative “house treatment” administered or suggested by unofficial therapists for patients who refuse vaccination or appeal to the no existence of SARS‐CoV‐2 infection. Sites suggesting the use of hydroxychloroquine (https://ippocrateorg.org/wp‐content/uploads/2020/11/IT‐Approccio‐alla‐terapia‐domiciliare‐Covid‐19‐aggiornamento‐2021‐08‐05.pdf), together with other bizarre medications, are still abundantly present on the web and extensively consulted. Therefore, offering evidence on the unfavorable profile of hydroxychloroquine such as that of the present study is not a tardy message. Relevant new safety information obtained for an older drug such as hydroxychloroquine must be considered and applied for more appropriate and safer use of hydroxychloroquine in patients with rheumatoid arthritis and lupus.

In conclusion, the results of present pharmacovigilance study, based on European data, may be added to the most recent scientific evidence to confirm the unfavorable safety profile of hydroxychloroquine in the treatment of COVID‐19 disease.

## CONFLICT OF INTEREST

All the authors declare no conflict of interest

## AUTHOR CONTRIBUTIONS

Substantial contributions to conception or design of the work (G.B., D.M., and NM) or the acquisition (G.B.), analysis (G.B., D.M., and N.M.), or interpretation of data for the work (N.M., G.B., and D.M.). Drafting of the work (G.B. and D.M.) or revising it critically for important intellectual content (N.M.). All authors approved the submitted final version to be published. All authors agree to be accountable for all aspects of the work in ensuring that questions related to the accuracy or integrity of any part of the work are appropriately investigated and resolved.

## Data Availability

The data that support the findings of this study are available from the corresponding author upon reasonable request.
